# Supplementary material for: UBB pseudogene 4 encodes functional ubiquitin variants
Source: Nat Commun. 2020 Mar 11;11:1306. doi: 10.1038/s41467-020-15090-6 (PMC7066184; doi:10.1038/s41467-020-15090-6)
Supplement: Supplementary file 1 — Supplementary Information [file 41467_2020_15090_MOESM1_ESM.pdf]

UBB Pseudogene 4 encodes functional ubiquitin variants

Dubois et al.

Supplementary Figure 1

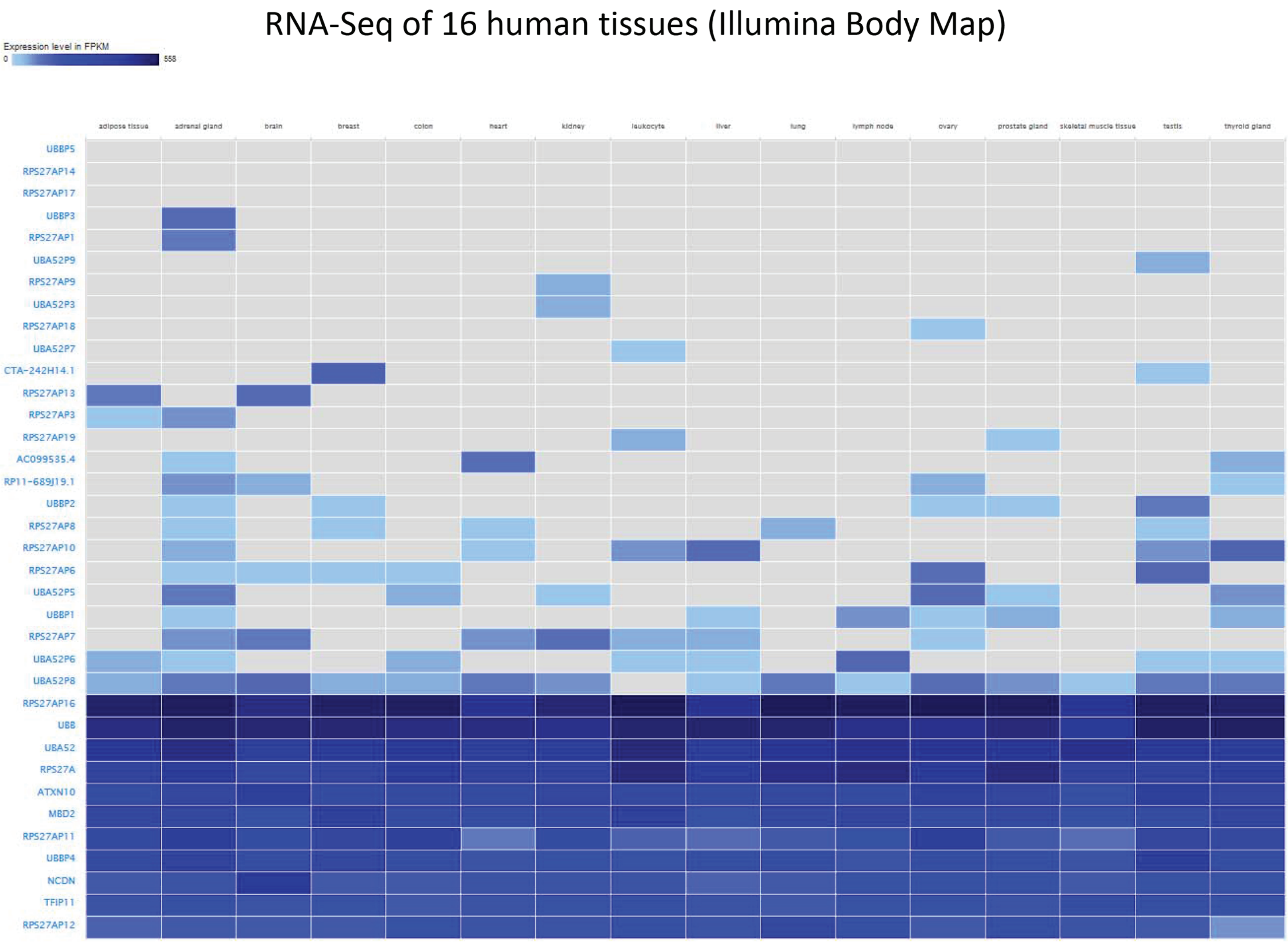

Supplementary Figure 1: Evidence of UBBP4 expression from Illumina Body Map. Expression levels analysis of *UBBP4* through RNASeq data available through the Illumina Body Map from 16 different tissues of all ubiquitin encoding genes and pseudogenes. Expression levels are shown in FPKM.

Supplementary Figure 2

RNA-Seq of 53 human tissues (Genotype-Tissue Expression Project (GTEx))

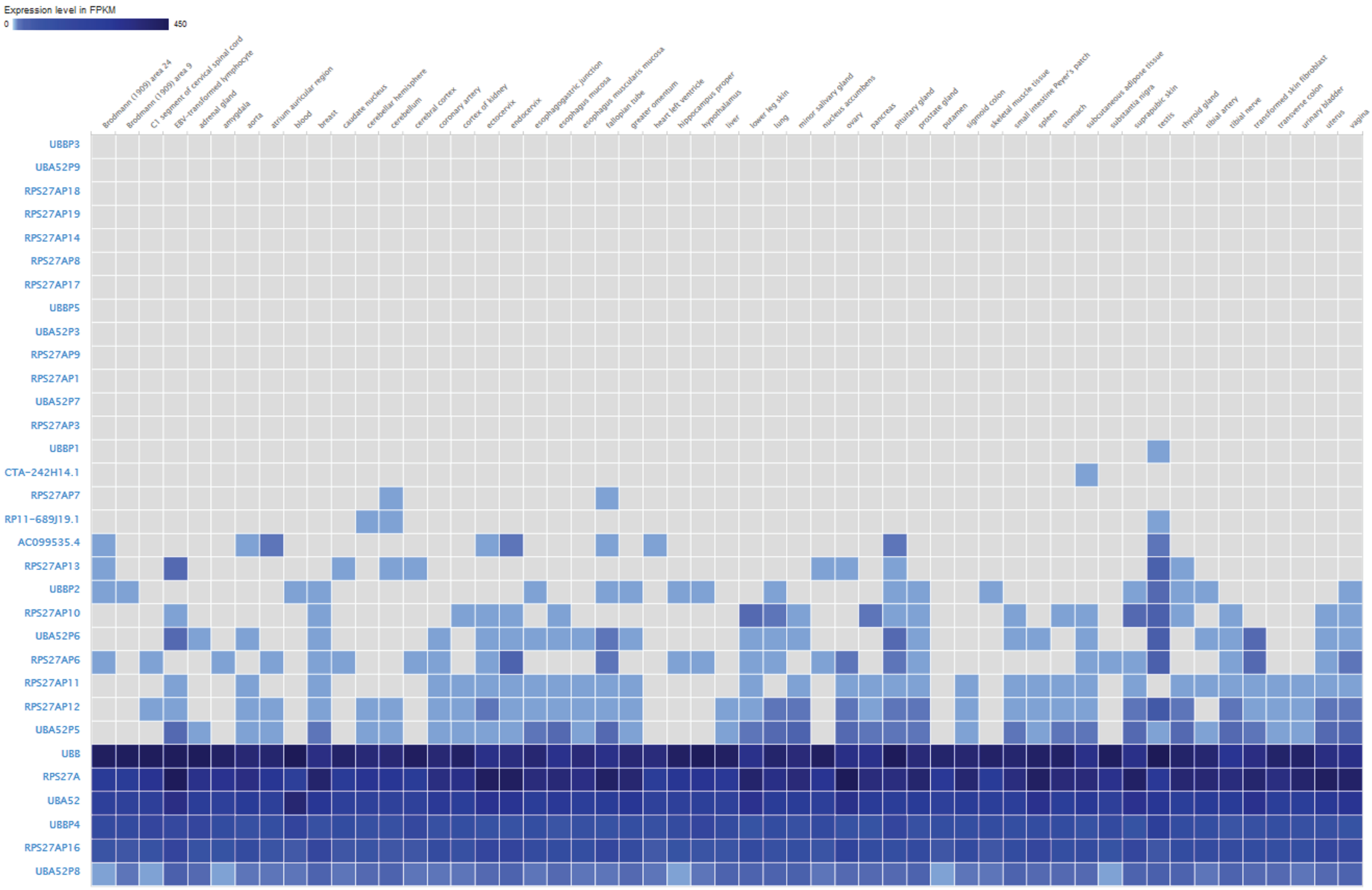

Supplementary Figure 2: Evidence of UBBP4 expression from the Genotype-Tissue Expression Project (GTEx).
Expression levels analysis of *UBBP4* through RNASeq data available through the GTEx Portal from 53 different tissues of all ubiquitin encoding genes and pseudogenes. Expression levels are shown in FPKM.

Supplementary Figure 3

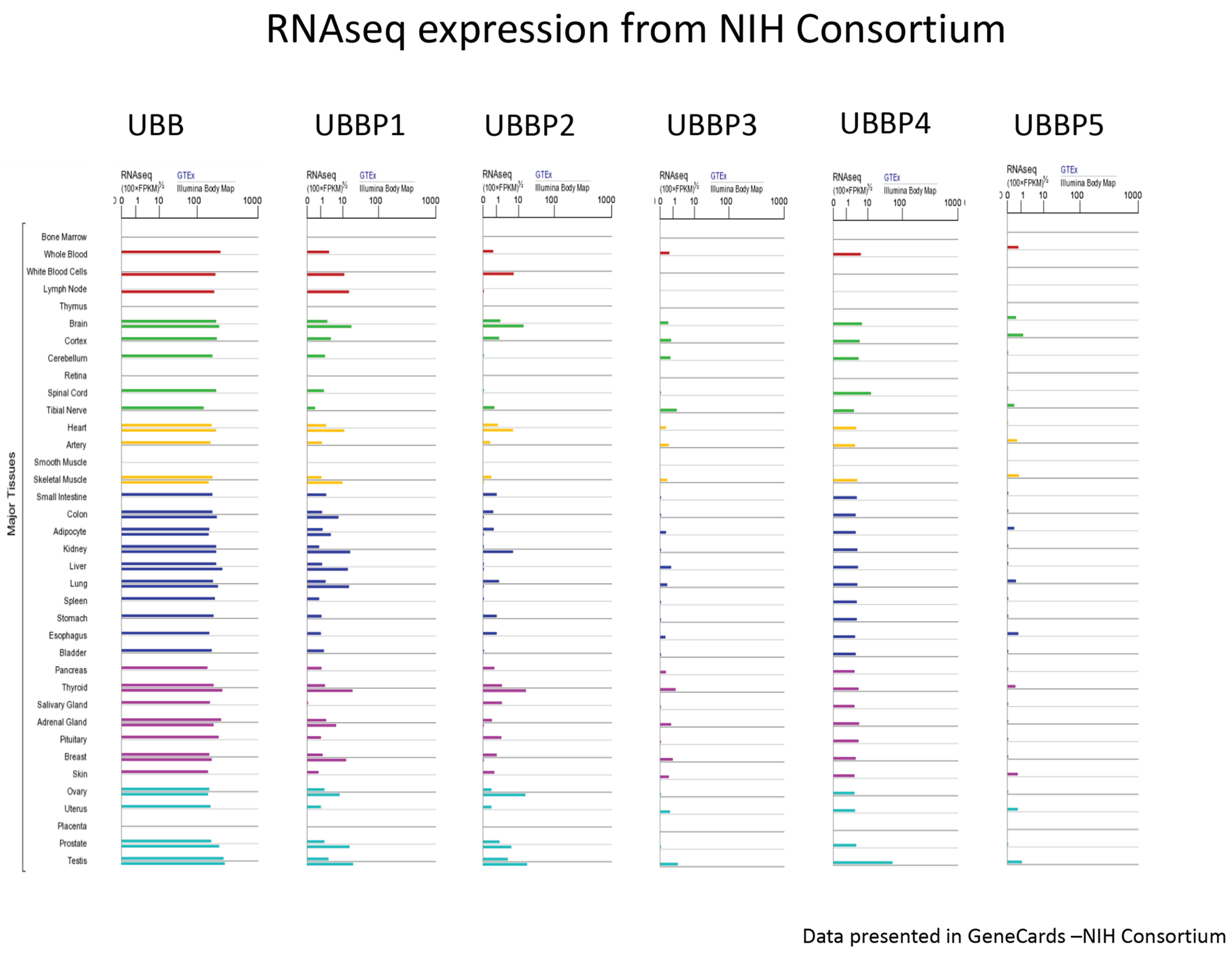

Supplementary Figure 3: Evidence of UBBP4 expression from the NIH Consortium. Expression levels analysis of *UBBP4* through RNASeq data available through the NIH Consortium and available through Genecards from 37 different major tissues of all ubiquitin encoding genes and pseudogenes. Expression levels are shown in FPKM.

Supplementary Figure 4

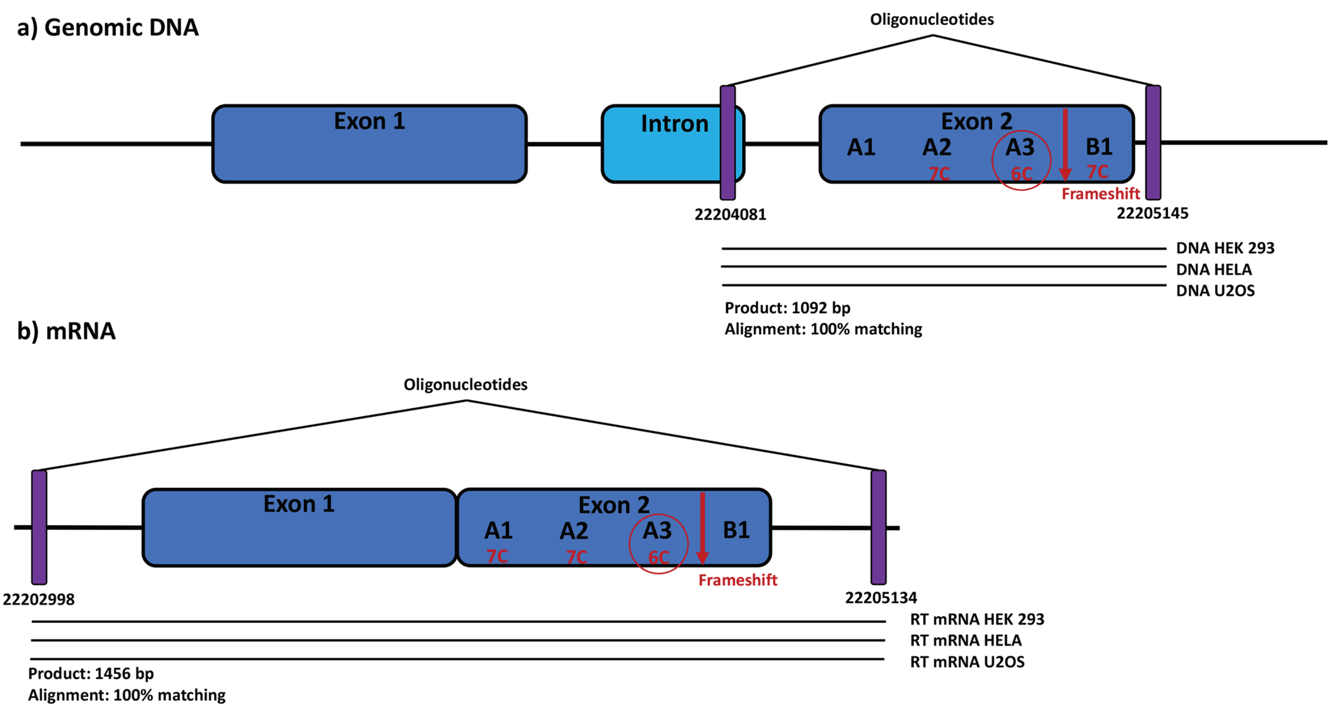

Supplementary Figure 4: Sequencing of UBBP4 genomic DNA and mRNA in HEK293, HeLa and U2OS cells.
a) Genomic DNA was extracted from HEK293, U2OS or HeLa cells. The genomic DNA specific to *UBBP4* was amplified by PCR using a first oligonucleotide within the intron, and a second oligonucleotide 3' of the exon 2 within the non-coding genomic region. **b)** RNA was isolated from HEK293, U2OS or HeLa cells. The isolated RNA was reversed transcribed using an oligo-dT. The reversed transcribed mRNA including both exons was amplified using oligonucleotides from the 5' to the 3' untranslated regions of the mRNA. Following PCR amplification of genomic DNA or reverse-transcribed RNA, the products were cloned into pUC19. A blue-white colony selection with X-galactosidase was performed, and ten clones for each cell line were then sequenced. All sequences were identical, and revealed the absence of the stop codon that was initially reported[1].

Supplementary Figure 5

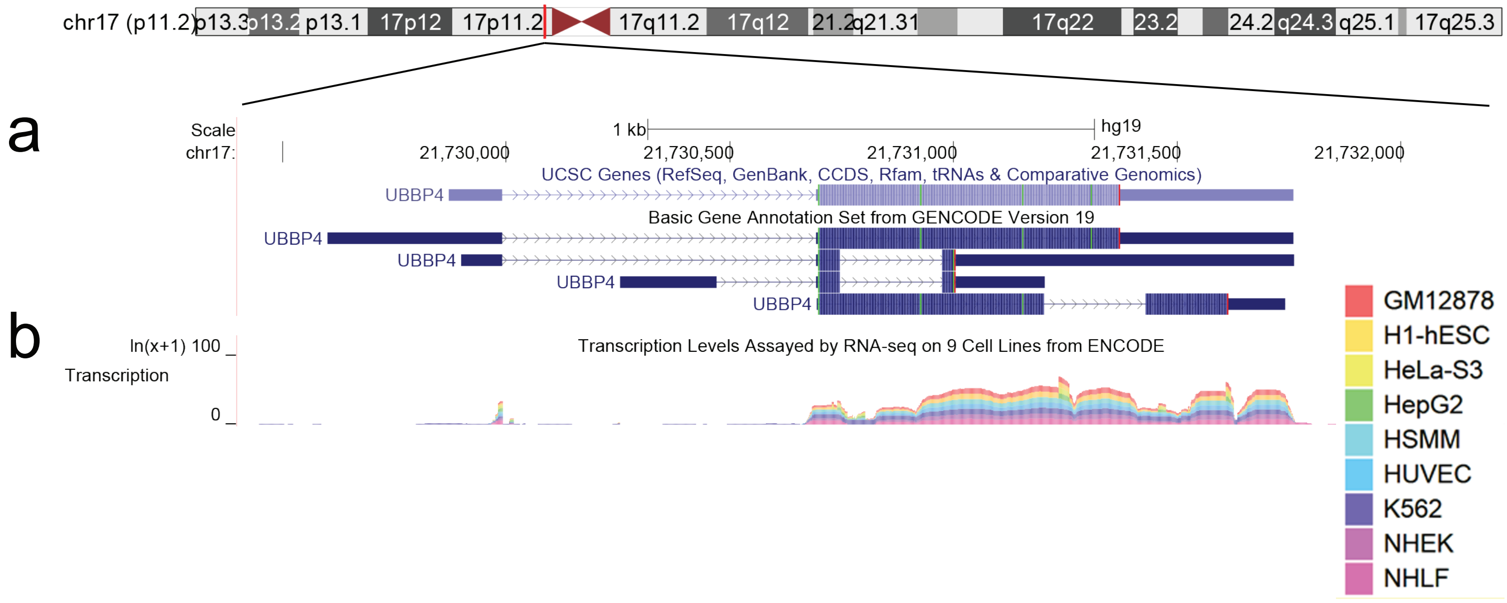

Supplementary Figure 5: Evidence of UBBP4 mRNA expression in nine different cell lines from ENCODE.

a) Localization of *UBBP4* genomic sequences on Chr 17. **b)** Analysis of RNA-Seq data from ENCODE indicates transcription covering both exons of the *UBBP4* gene. Colours indicate nine different cell lines (GM12878, H1-hESC, HeLa-S3, HepG2, HSMM, HUVEC, K562, NHEK and NHLF).

Supplementary Figure 6

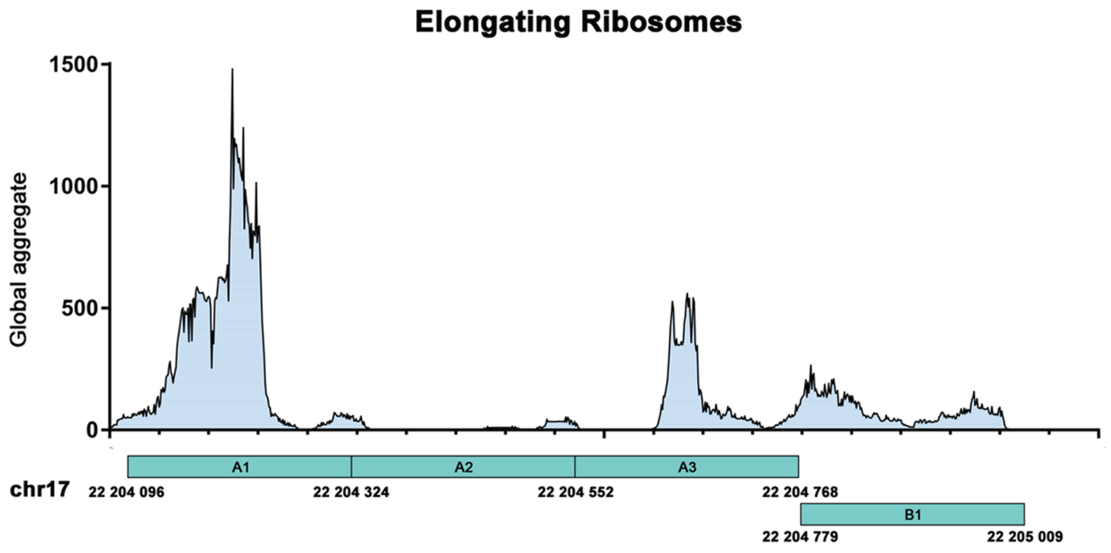

Supplementary Figure 6: Evidence of UBBP4 translation.
Ribosome profiling uses high throughput sequencing to identify mRNA fragments that are protected by elongating ribosomes. The presence of ribosomes on the mRNA of *UBBP4* from ribosome profiling databases (GWIPS-viz) identifies reads throughout both *UBBP4* reading frames, covering Ubbp4^{A1}, ^{A2}, ^{A3} and ^{B1}.

Supplementary Figure 7

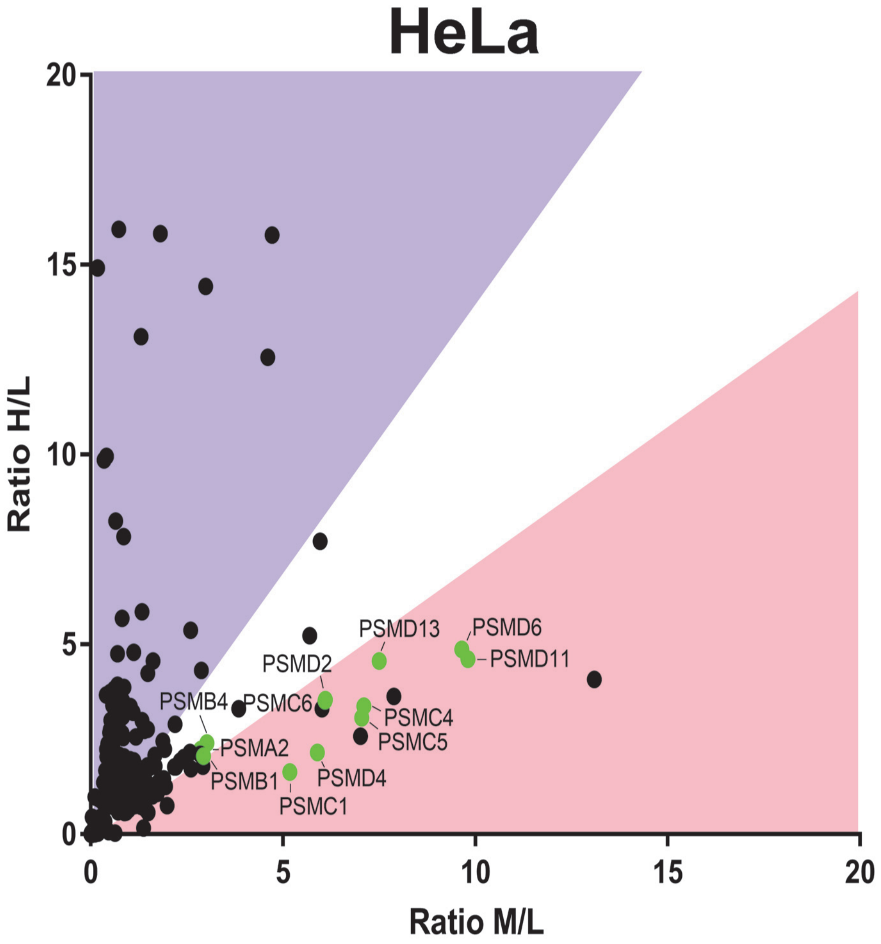

Supplementary Figure 7: Identification of proteins interacting with Ub and Ub^{KEKS}
SILAC-labelled HeLa cells were transfected with empty vector (pcDNA, light), HA-Ub (medium) or HA-Ub^{KEKS} (heavy) and lysed under non-denaturing conditions. Following identification and quantification by mass spectrometry, the average ratios identify proteins co-immunoprecipitating with Ub (M/L) or Ub^{KEKS} (H/L). Subunits of the proteasome are shown in green.

Supplementary Figure 8

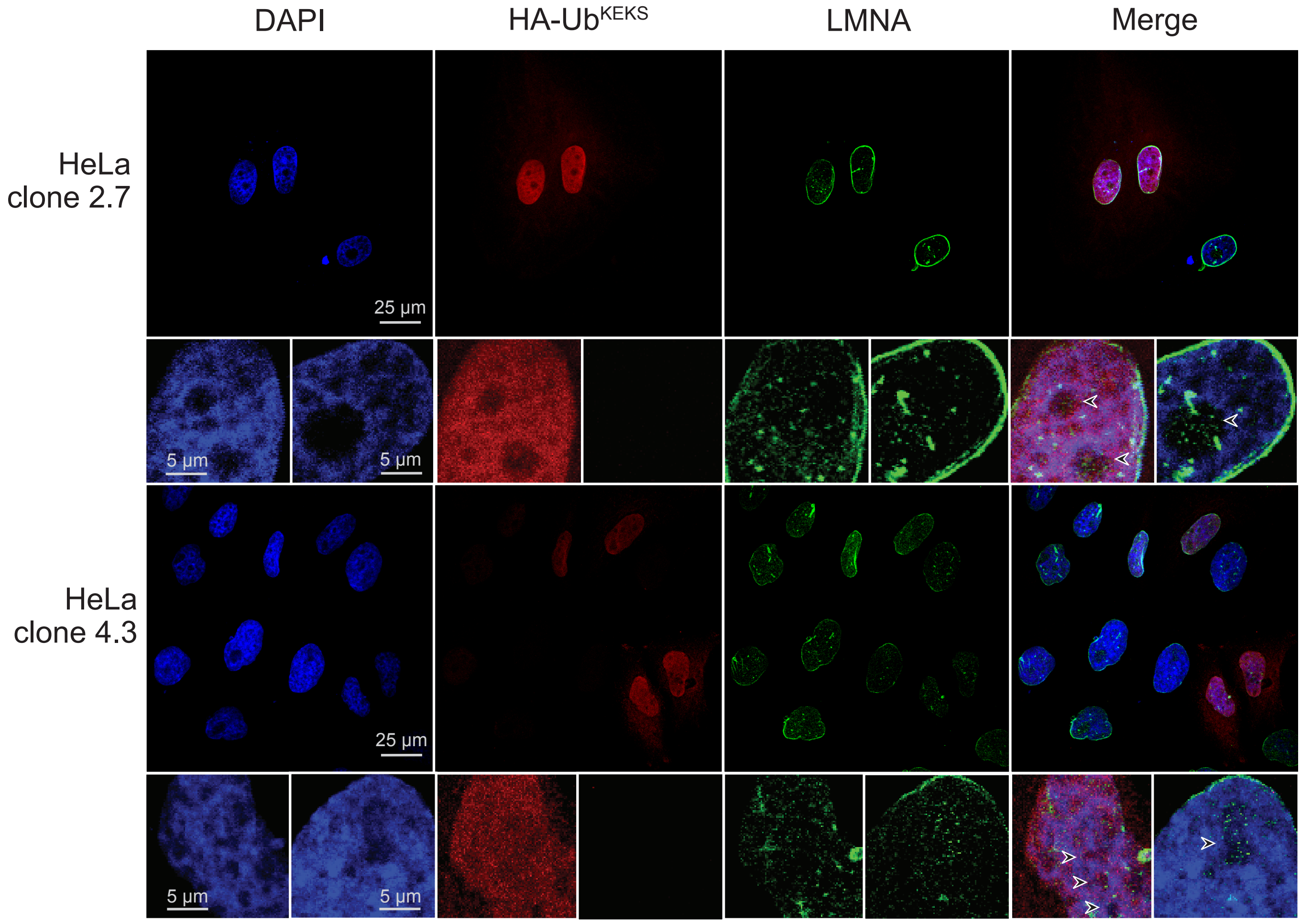

Supplementary Figure 8: Lamin A nucleolar localization was rescued in HeLa Ub^{KEKS} KO cells (clone 4.3 and 2.7) with transient transfection of HA-Ub^{KEKS}.

HeLa cells KO for Ub^{KEKS} (clones 2.7 and 4.3) were transfected with a plasmid encoding HA-tagged Ub^{KEKS}. 72 hours post-transfection, cells were fixed and labelled for immunofluorescence microscopy with a HA antibody (red), or a Lamin A antibody (green). The nuclei were stained with DAPI (n=3 independent experiments).

Supplementary Figure 9

a) Product ion intensities of Ub peptide

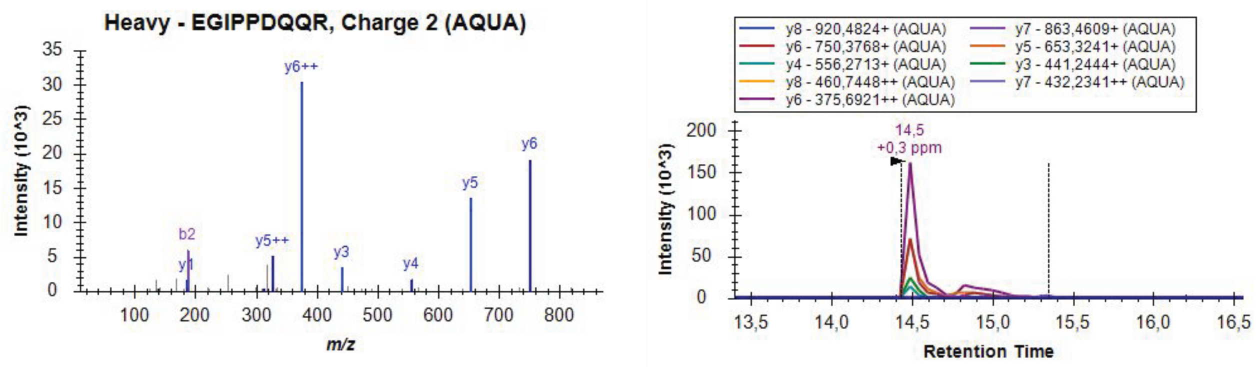

Product ion intensities of Ub^{KEKS} peptide

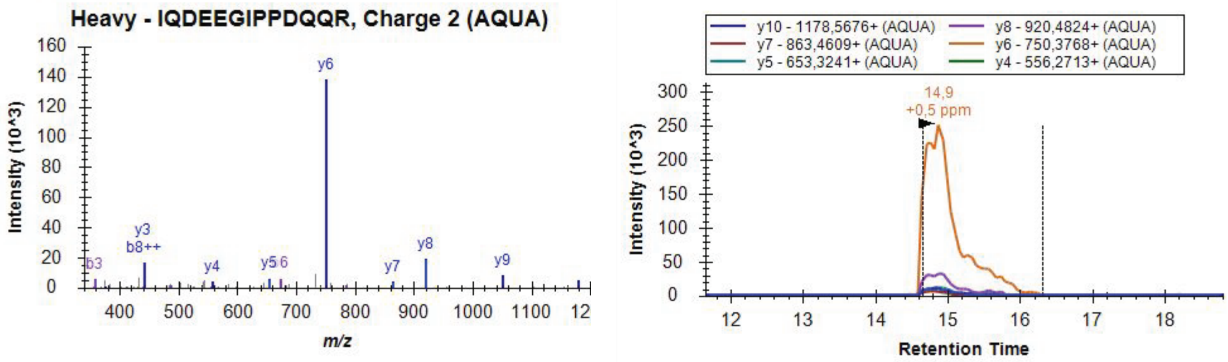

b) Ub^{KEKS} heavy peptide alone | Hela WT | Hela WT with HA-Ub^{KEKS} overexpression

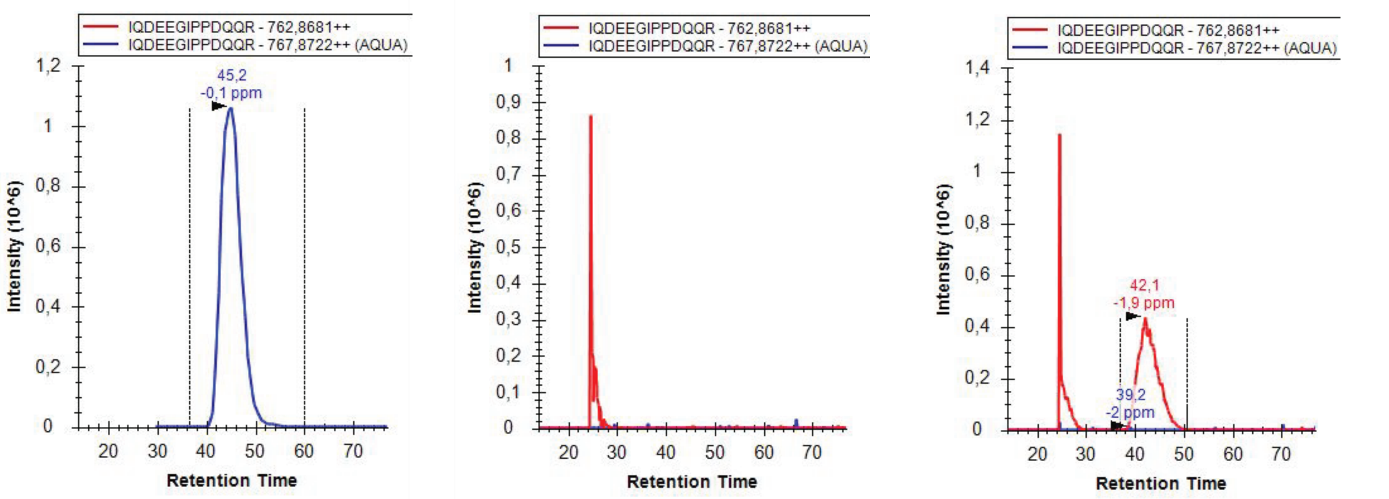

c) Ub^{KEKS}peptides identified in LMNA pulldown assay | Ub^{KEKS} peptides identified in LMNB2 pulldown assay

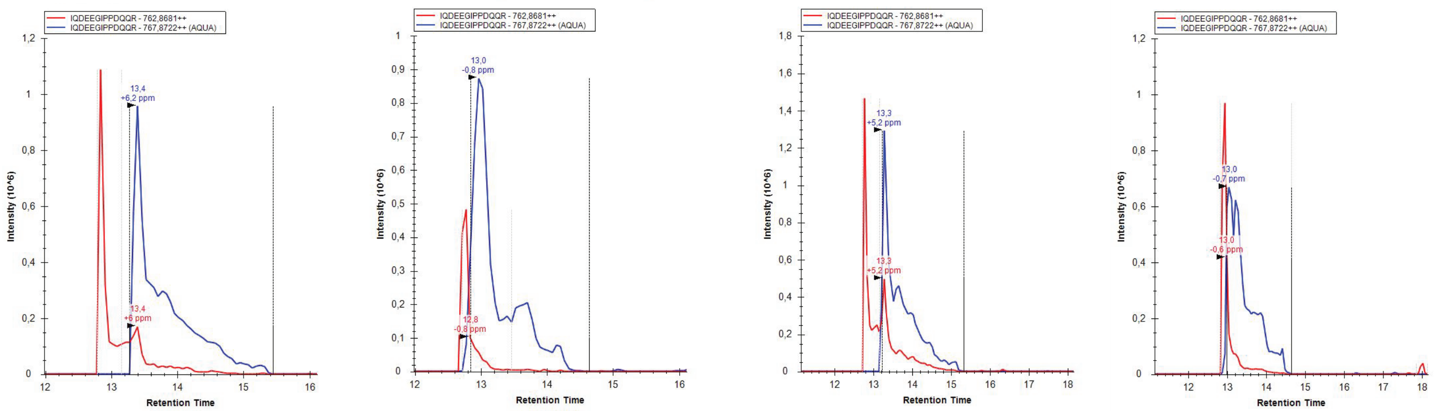

HeLa WT | HeLa 4.3 | HeLa WT | HeLa 4.3

Supplementary Figure 9: Optimization of Ub and Ub^{KEKS} quantification and detection of Ub^{KEKS} peak.

a) Product ion intensities of Ub and Ub^{KEKS} peptides (m/z and retention times). The most intense ion for Ub y6^{++} and for Ub^{KEKS} y6^{+} were selected to be used for quantification. b) Ub^{KEKS} peak identification test using a 50cm column with a 120min run. Runs were performed using Ub^{KEKS} heavy peptide alone, HeLa total cell extract or Hela overexpressed total cell extract (cells were transfected with HA-Ub^{KEKS} plasmid for 48h). Retention times of the heavy peptide and the two different peaks coming from the Hela or HeLa overexpressed cells were compared and the peak present only in transfected cells was confirmed to be the peak corresponding to Ub^{KEKS} light peptides. The peak eluting before was regarded as non-specific signal. c) Chromatogram Ub^{KEKS} in WT and Ub^{KEKS} KO (clone 4.3) HeLa cells in LMNA and LMNB2 pulldown assays. Blue line represents heavy (AQUA) peptide, red line represents endogenous peptides with m/z values indicated in the box above chromatograms. Dotted lines indicate peak boundaries with black arrows showing peaks. Peak values with mass error are indicated above each peak. Within peak boundaries the highest measured value for both heavy and light peptides were recorded. However, due to the limitations of this type of measurement peak trailing appearing from the non-specific signal bleeding into the measurement area corresponded to recorded values even in the absence of a real peak.

Supplementary Figure 10

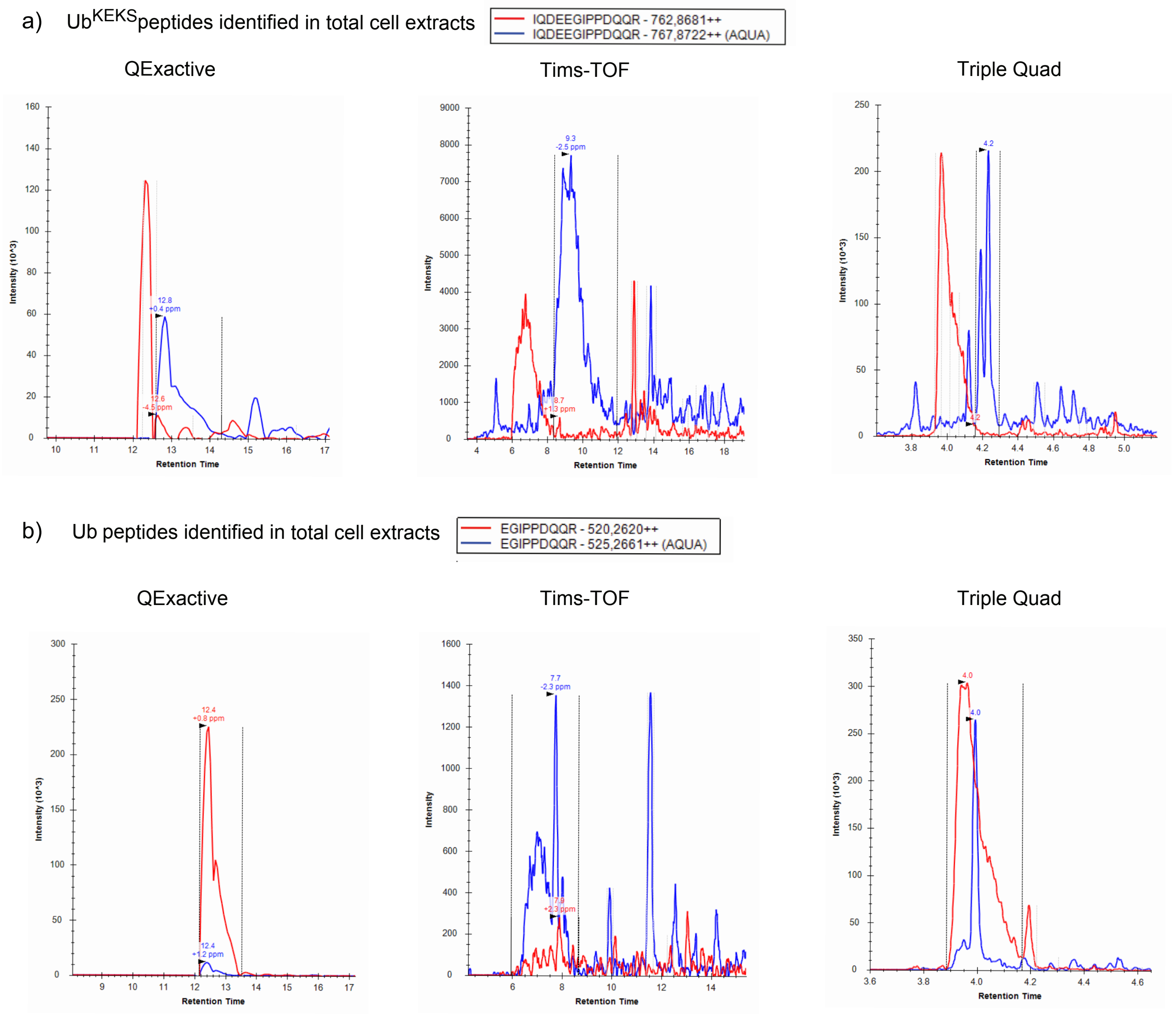

Supplementary Figure 10: Absolute quantification of Ub and Ub^{KEKS} in total cell extracts.
a-b) Exponentially growing HeLa WT were harvested and lysed. Following tryptic digestion, the Heavy Arginine (U-13C6, 15N4; mass difference: +10 Da) labelled AQUA Ub (EGIPPDQQR) and UbKEKS (IQDEEGIPPDQQR) peptides were spiked into the samples at a final concentration of 1.66 fmol/µl. The peptides were analysed by mass spectrometers using a PRM method with an inclusion list containing the m/z values corresponding to the monoisotopic form of the heavy and light peptides of Ub (520.2/ 525.2) and Ub^{KEKS} (762.8/ 767.8). For quantification, the most intense fragment ion (y6) was used for both peptides. The analysis were performed on an OrbiTrap QExactive (Thermo Fisher), a TimsTOF Pro (Bruker Daltonics) and a LCMS-8060 (Shimadzu). The quantification using SkyLine is presented in Supplementary Table 4.

Supplementary Figure 11

a

altprot_accession	Pseudogene	single pep	reads_tis	gene_symbol	transcript_accession	Motif-RGG
IP_634590.1		0	0	AC008072.1	ENST00000434254	
IP_639873.1	RPS27A pseudogene 7	2	0	AC079807.3	ENST00000422792	
IP_584271.1		1	0	CTA-242H14.1	ENST00000466070	
IP_602299.1	RPS27A pseudogene 10	1	0	CTD-2024I7.1	ENST00000496103	
IP_689793.1	RPS27A pseudogene 19	1	0	CTD-3214H19.12	ENST00000597285	RGG
IP_592206.1	RPS27A pseudogene 11	1	0	RP11-367G18.2	ENST00000402798	RGG
IP_592206.1	RPS27A pseudogene 11	1	0	RP11-367G18.2	ENST00000402798	RGG
IP_722705.1	RPS27A pseudogene 16	0	235	RP11-51O6.1	ENST00000492222	
IP_750298.1	UBA52 pseudogene 2	0	0	RP11-689J19.1	ENST00000480068	
IP_710165.1		0	0	RP11-963H4.2	ENST00000478739	
IP_761918.1		2	0	RP3-432I18.1	ENST00000546846	
IP_579682.1	UBA52 pseudogene 12	0	0	RP4-814D15.2	ENST00000429552	
IP_713422.1	RPS27A pseudogene 1	0	237	RPS27AP1	ENST00000475545	RGG
IP_663056.1	RPS27A pseudogene 2	0	0	RPS27AP2	ENST00000457576	
IP_662482.1	RPS27A pseudogene 3	0	0	RPS27AP3	ENST00000453803	
IP_564850.1	UBA52 pseudogene 6	2	0	UBA52P6	ENST00000399822	RGG
IP_723668.1	UBA52 pseudogene 8	0	0	UBA52P8	ENST00000498379	
IP_636378.1	UBB pseudogene 1	0	448	UBBP1	ENST00000392399	
IP_636379.1	UBB pseudogene 1	0	142	UBBP1	ENST00000392399	
IP_668239.1	UBB pseudogene 2	0	442	UBBP2	ENST00000376781	
IP_636103.1	UBB pseudogene 3	0	0	UBBP3	ENST00000445497	RGG

b

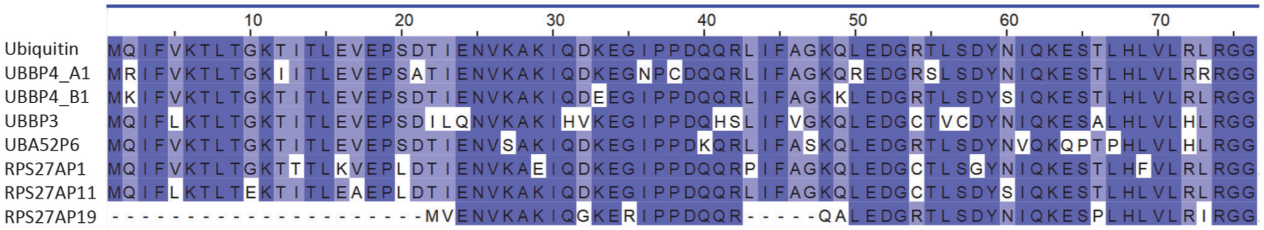

Supplementary Figure 11: Several ubiquitin variants are produced from ubiquitin pseudogenes.
a) Large scale proteomic experiments and ribosome profiling uncovers evidence at the protein level for several ubiquitin pseudogenes producing different ubiquitin variants[2]. Altprot_accession: in the OpenProt proteogenomic resource[2], novel alternative proteins, including those coded by pseudogenes are given IP_accession numbers. Single pep: number of peptides unique (i.e. specific) for the protein coded by the corresponding pseudogene. Reads_tis: ribosome profiling reads at the translation initiation site. **b)** The human ubiquitin variants ending with a di-glycine at the C-terminus are compared for their amino acid sequences.

Supplementary Figure 12. Full scan of western blots with molecular weight

Figure 2c

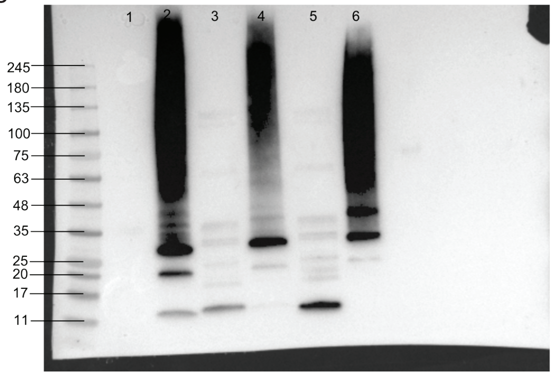

Use of αHA:
1) MOCK cells
2) HA-Ub
3) HA-UBBP4^{A1}
4) HA-UBBP4^{A2}
5) HA-UBBP4^{A3}
6) HA-UBBP4^{B1}

Figure 2d

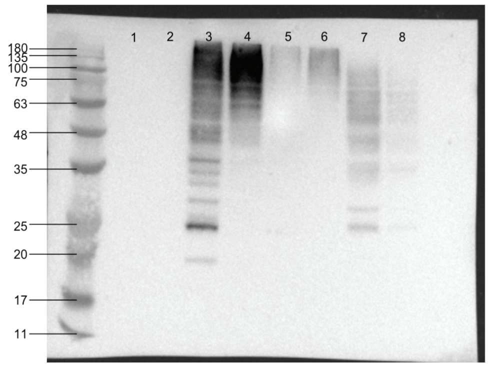

Use of αHA:
1) pcDNA (without Mg132)
2) pcDNA (with Mg132)
3) HA-Ub (without Mg132)
4) HA-Ub (with Mg 132)
5) HA-Ubbp4^{A2} (without Mg132)
6) HA-Ubbp4^{A2} (with Mg132)
7) HA-Ubbp4^{B1} (without Mg132)
8) HA-Ubbp4^{B1} (with Mg132)

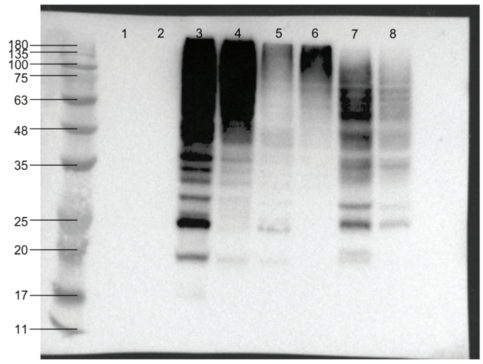

Use of αHA:
1) pcDNA (without Mg132)
2) pcDNA (with Mg132)
3) HA-Ub (without Mg132)
4) HA-Ub (with Mg 132)
5) HA-Ubbp4^{A2} (without Mg132)
6) HA-Ubbp4^{A2} (with Mg132)
7) HA-Ubbp4^{B1} (without Mg132)
8) HA-Ubbp4^{B1} (with Mg132)

Figure 3a

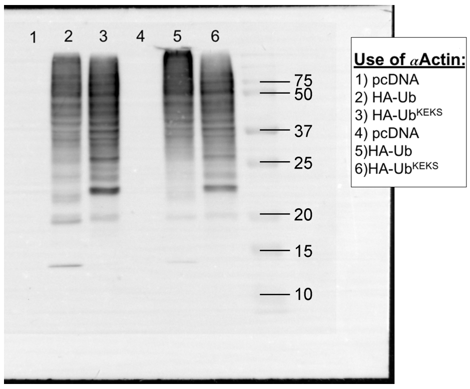

Use of αActin:
1) pcDNA
2) HA-Ub
3) HA-Ub^{KEKS}
4) pcDNA
5)HA-Ub
6)HA-Ub^{KEKS}

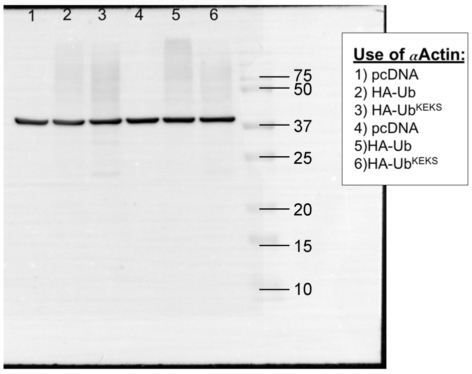

Use of αActin:
1) pcDNA
2) HA-Ub
3) HA-Ub^{KEKS}
4) pcDNA
5)HA-Ub
6)HA-Ub^{KEKS}

Figure 4a

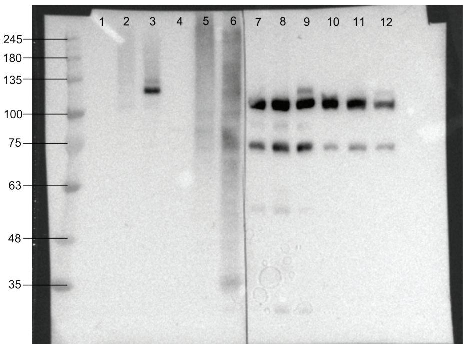

Use of αHA:
1) LMNA-GFP + pcDNA (IP-GFP)
2) LMNA-GFP + HA-Ub (IP-GFP)
3) LMNA-GFP + HA-Ub^{KEKS} (IP-GFP)
4) LMNA-GFP + pcDNA (Extract)
5) LMNA-GFP + HA-Ub (Extract)
6) LMNA-GFP + HA-Ub^{KEKS} (Extract)

Use of αGFP:
7) LMNA-GFP + pcDNA (IP-GFP)
8) LMNA-GFP + HA-Ub (IP-GFP)
9) LMNA-GFP + HA-Ub^{KEKS} (IP-GFP)
10) LMNA-GFP + pcDNA (Extract)
11) LMNA-GFP + HA-Ub (Extract)
12) LMNA-GFP + HA-Ub^{KEKS} (Extract)

Figure 4b

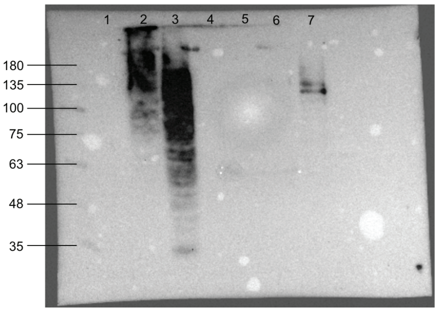

Use of αHA :
1) LMNB2-GFP + pcDNA (Extract)
2) LMNB2-GFP + HA-Ub (Extract)
3) LMNB2-GFP + HA-Ub^{KEKS} (Extract)
4) *Empty*
5) LMNB2-GFP + pcDNA (IP-GFP)
6) LMNB2-GFP + HA-Ub (IP-GFP)
7) LMNB2-GFP + HA-Ub^{KEKS} (IP-GFP)

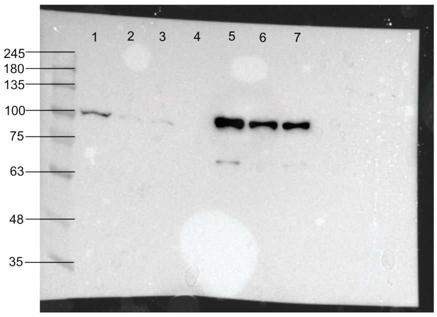

Use of αGFP :
1) LMNB2-GFP + pcDNA (Extract)
2) LMNB2-GFP + HA-Ub (Extract)
3) LMNB2-GFP + HA-Ub^{KEKS} (Extract)
4) *Empty*
5) LMNB2-GFP + pcDNA (IP-GFP)
6) LMNB2-GFP + HA-Ub (IP-GFP)
7) LMNB2-GFP + HA-Ub^{KEKS} (IP-GFP)

Supplementary Table 1. Overview of human ubiquitin-coding genes and their pseudogenes

Human	Ubiquitin-coding genes	RPS27A	UBA52	UBB	UBC	
	**Annotated Pseudogenes**	RPS27AP1	UBA52P1	UBBP1	AL596327.1	
		RPS27AP2	UBA52P2	UBBP2	AC108676.2	
		RPS27AP3	UBA52P3	UBBP3		
		RPS27AP4	UBA52P4	UBBP4		
		RPS27AP5	UBA52P5	UBBP5		
		RPS27AP6	UBA52P6			
		RPS27AP7	UBA52P7			
		RPS27AP8	UBA52P8			
		RPS27AP9	UBA52P9			
		RPS27AP10				
		RPS27AP11				
		RPS27AP12				
		RPS27AP13				
		RPS27AP14				
		RPS27AP15				
		RPS27AP16				
		RPS27AP17				
		RPS27AP18				
		RPS27AP19				
						**Total**
	**Number of pseudogenes**	17	6	5	2	52

Supplementary Table 2. Oligonucleotides used in this study

UBBP4 CRISPR/Cas Oligonucleotides

sgRNA	target sequence	Top oligo (5'-3')	Bottom oligo (5'-3')
**#30**	GCAGAGCGCAAATTTGTGCA	CACCGCAGAGCGCAAATTTGTGCA	AAACTGCACAAATTTGCGCTCTGC
**#12**	GCAATCCCTGTGACCAGCAG	CACCGCAATCCCTGTGACCAGCAG	AAACCTGCTGGTCACAGGGATTGC
**#5**	GCAGGCAAGAAGTTGGAAGA	CACCGCAGGCAAGAAGTTGGAAGA	AAACTCTTCCAACTTCTTGCCTGC

gblock oligonucleotides

**HA-Ub**	GACTCGAGATGGGCTACCCCTATGATGTGCCTGACTACGCAGATCTCAATGGTGGTGGTGGTGGGTCGACCATGCAGATCTTCGTGAAGACCCTGACTGGTAAGACCATCACTCTCGAAGTGG<br>AGCCGAGTGACACCATTGAGAATGTCAAGGCAAAGATCCAAGACAAGGAAGGCATCCCTCCTGACCAGCAGAGGTTGATCTTTGCTGGGAAACAGCTGGAAGATGGACGCACCCTGTCTGAC<br>TACAACATCCAGAAAGAGTCCACCCTGCACCTGGTCCTCCGTCTCAGAGGTGGTTGAGGATCCGA
**HA-Ub^{KEKS}**	GACTCGAGATGGGCTACCCCTATGATGTGCCTGACTACGCAGATCTCAATGGTGGTGGTGGTGGGTCGACCATGAAGATCTTCGTGAAGACCCTGACTGGAAAGACCATCACCCTGGAGGTGG<br>AGCCCAGTGACACCATCGAAAATGTGAAAGCCAAGATCCAGGATGAAGAAGGCATCCCCCCCGATCAGCAGAGGCTCATCTTTGCAGGCAAGAAGTTGGAAGATGGCCGCACTCTTTCTGACT<br>ACAGCATCCAGAAAGAGTCGACCCTGCACCTGGTCCTGCGCCTGAGGGGTGGCTGTTAAGGATCCGA

Oligonucleotides for PCR

Genes	Forward primer	Reverse primer
**LMNA**	GGGGACAAGTTTGTACAAAAAAGCAGGCTTCatggagaccccgtcccag	GGGGACCACTTTGTACAAGAAAGCTGGGTGcatgatgctgcagttctgggg
**LMNB2**	GGGGACAAGTTTGTACAAAAAAGCAGGCTTCatgagcccgccgagcccg	GGGGACCACTTTGTACAAGAAAGCTGGGTGcatcacgtagcagcctcttgaggt

Supplementary References

1 Cowland, J. B., Wiborg, O. & Vuust, J. Human ubiquitin genes: one member of the UbB gene subfamily is a tetrameric non-processed pseudogene. *FEBS Lett* **231**, 187-191 (1988).

2 Brunet, M. A. *et al.* OpenProt: a more comprehensive guide to explore eukaryotic coding potential and proteomes. *Nucleic Acids Res* **47**, D403-D410, doi:10.1093/nar/gky936 (2019).
