# Supplementary material for: UBB pseudogene 4 encodes functional ubiquitin variants
Source: Nat Commun. 2020 Mar 11;11:1306. doi: 10.1038/s41467-020-15090-6 (PMC7066184; doi:10.1038/s41467-020-15090-6)
Supplement: Supplementary file 3 — Description of Additional Supplementary Files [file 41467_2020_15090_MOESM3_ESM.docx]

**Description of Additional Supplementary Files**

**File Name: Supplementary Data 1**

**Description:** Quantitative mass spectrometry identification of proteins modified by Ub and UbKEKS or interacting with Ub and UbKEKS SILAC-labelled HeLa cells were transfected with empty vector (pcDNA, light), HA-Ub (medium) or HA-UbKEKS (heavy) and lysed under non-denaturing conditions (high salt, HS) or denaturing conditions (SDS). Following identification and quantification by mass spectrometry, the average of triplicate experiments identify proteins coimmunoprecipitating with Ub (M/L) or UbKEKS (H/L).

**File Name: Supplementary Data 2**

**Description:** Absolute quantification of Ub and UbKEKS in total cell extracts. Quantification results using SkyLine on HeLa whole cell extracts for Ub and UbKEKS, using an OrbiTrap QExactive (Thermo Fisher), a TimsTOF Pro (Bruker Daltonics) and a LC-8060 (Shimadzu).

**File Name: Supplementary Data 3**

**Description:** Absolute quantification of Ub and UbKEKS on Lamin A and Lamin B2 Quantification using SkyLine of immunoprecipitated GFP-Lamin A or GFP-Lamin B2 expressed in wild-type HeLa cells, or HeLa cells KO for UbKEKS (clones 2.7 and 4.3) (n=4).
