# Supplementary material for: UBB pseudogene 4 encodes functional ubiquitin variants
Source: Nat Commun. 2020 Mar 11;11:1306. doi: 10.1038/s41467-020-15090-6 (PMC7066184; doi:10.1038/s41467-020-15090-6)
Supplement: Supplementary file 7 — Reporting Summary [file 41467_2020_15090_MOESM7_ESM.pdf]

nature research

Corresponding author(s): Francois-Michel Boisvert

Last updated by author(s): Feb 13, 2020

Reporting Summary

Nature Research wishes to improve the reproducibility of the work that we publish. This form provides structure for consistency and transparency in reporting. For further information on Nature Research policies, see Authors & Referees and the Editorial Policy Checklist .

Statistics

For all statistical analyses, confirm that the following items are present in the figure legend, table legend, main text, or Methods section.

n/a	Confirmed	
☐	☒	The exact sample size (n) for each experimental group/condition, given as a discrete number and unit of measurement
☐	☒	A statement on whether measurements were taken from distinct samples or whether the same sample was measured repeatedly
☐	☒	The statistical test(s) used AND whether they are one- or two-sided<br>*Only common tests should be described solely by name; describe more complex techniques in the Methods section.*
☒	☐	A description of all covariates tested
☐	☒	A description of any assumptions or corrections, such as tests of normality and adjustment for multiple comparisons
☐	☒	A full description of the statistical parameters including central tendency (e.g. means) or other basic estimates (e.g. regression coefficient) AND variation (e.g. standard deviation) or associated estimates of uncertainty (e.g. confidence intervals)
☒	☐	For null hypothesis testing, the test statistic (e.g. F, t, r) with confidence intervals, effect sizes, degrees of freedom and P value noted<br>*Give P values as exact values whenever suitable.*
☒	☐	For Bayesian analysis, information on the choice of priors and Markov chain Monte Carlo settings
☒	☐	For hierarchical and complex designs, identification of the appropriate level for tests and full reporting of outcomes
☒	☐	Estimates of effect sizes (e.g. Cohen's d, Pearson's r), indicating how they were calculated

Our web collection on statistics for biologists contains articles on many of the points above.

Software and code

Policy information about availability of computer code

Data collection	Xcalibur version 2.2 SP1.48 was used for mass spectrometry acquisition of immunoprecipitations. PRM quantification data collection was done with Xcalibur version 3.1.66.10. LabSolution v5.93 software was used for acquisition by the Shimadzu LCMS-8060.
Data analysis	Identification and quantification of proteins identified by mass spectrometry was done using the MaxQuant software version 1.5.2.8. The quantification of nucleoli area was achieved with CellProfiler 3.1.8. FACS analysis was achieved with the FlowJo software 10.6.1. Statistical analysis was done with GraphPad Prism 8.

For manuscripts utilizing custom algorithms or software that are central to the research but not yet described in published literature, software must be made available to editors/reviewers. We strongly encourage code deposition in a community repository (e.g. GitHub). See the Nature Research guidelines for submitting code & software for further information.

Data

Policy information about availability of data

All manuscripts must include a data availability statement. This statement should provide the following information, where applicable:

- Accession codes, unique identifiers, or web links for publicly available datasets
- A list of figures that have associated raw data
- A description of any restrictions on data availability

The mass spectrometry proteomics data have been deposited to the ProteomeXchange Consortium via the PRIDE partner repository with the dataset identifier PXD014318 (Figure 3).

Field-specific reporting

Please select the one below that is the best fit for your research. If you are not sure, read the appropriate sections before making your selection.

- [x] Life sciences
- [ ] Behavioural & social sciences
- [ ] Ecological, evolutionary & environmental sciences

For a reference copy of the document with all sections, see nature.com/documents/nr-reporting-summary-flat.pdf

Life sciences study design

All studies must disclose on these points even when the disclosure is negative.

Sample size	Mass spectrometry data was performed in biological triplicates to allow statistical analyses (n=3). Growth curves were performed by FACS (n=4). Measurements of nucleoli was performed in biological triplicates (n=3). Minimal n=3 sizes were chosen when the data showed low variability between the samples.
Data exclusions	No data was excluded.
Replication	Every other experiments in the article were performed at least twice, and all the findings were reproduced.
Randomization	Cells used for immunofluorescence imaging and measurements were selected randomly for the quantification of nucleoli size and lamin localization. There was no need for randomization.
Blinding	Blinding was not possible as experimental conditions were evident from the image data and the initial observations were conclusive.

Reporting for specific materials, systems and methods

We require information from authors about some types of materials, experimental systems and methods used in many studies. Here, indicate whether each material, system or method listed is relevant to your study. If you are not sure if a list item applies to your research, read the appropriate section before selecting a response.

Materials & experimental systems

n/a	Involved in the study	
[ ]	[x]	Antibodies
[ ]	[x]	Eukaryotic cell lines
[x]	[ ]	Palaeontology
[x]	[ ]	Animals and other organisms
[x]	[ ]	Human research participants
[x]	[ ]	Clinical data

Methods

n/a	Involved in the study	
[x]	[ ]	ChIP-seq
[ ]	[x]	Flow cytometry
[x]	[ ]	MRI-based neuroimaging

Antibodies

Antibodies used

1) GFP-Trap Agarose antibodies (Chromotek, gta-20).
2) anti-GFP (Roche #11814460001)
3) LaminA (Abcam #ab133256)
4) HA-tag (Invitrogen #26183)
5) Nucleolin (Abcam #136649)
6) Goat anti-mouse AlexaFluor 488 (Invitrogen #A11001)
7) Goat anti-rabbit AlexaFluor 546 (Invitrogen #A11008)
8) HA-tag (12CA5 monoclonal antibody, Hybridoma grown from ATCC)
9) actin antibody (Sigma #A5441)

Validation

1) https://chromotek.com/products/detail/product-detail/gfp-trap-agarose/
2) Validated in WB and IP. Species are not relevant as this antibody recognizes a tag. Cited in 6 publication(s) in the manufacturer's application sheet.
3) Validated in WB, IHC, Flow Cyt, ICC/IF and tested in Mouse, Rat, Human. Cited in 4 publication(s).
4) Validated in WB, IHC, IP and ChIP. Species are not relevant as this is a tag. Cited in 29 publication(s).
5) Validated in WB, IHC, Flow Cyt, ICC/IF and tested in Mouse, Rat, Human. Cited in 86 publication(s).
6) Validated for immunofluorescence. Species are not relevant as this is a secondary antibody. Cited in 2981 publication(s) by the manufacturer.
7) Validated for immunofluorescence. Species are not relevant as this is a secondary antibody. Cited in 95 publication(s) by the manufacturer.

8) Validated for WB and IP. Species are not relevant as this is a tag. This is a historical hybridoma that has been used by thousands of laboratories. We grew the hybridoma, purified the antibody and tested the reactivity.

Eukaryotic cell lines

Policy information about cell lines

Cell line source(s)	HEK293, HeLa and U2OS are from ATCC.
Authentication	None of the cell lines used were authenticated.
Mycoplasma contamination	All cell lines were tested and are negative for mycoplasma.
Commonly misidentified lines (See ICLAC register)	Not applicable.

Flow Cytometry

Plots

Confirm that:

- [x] The axis labels state the marker and fluorochrome used (e.g. CD4-FITC).
- [x] The axis scales are clearly visible. Include numbers along axes only for bottom left plot of group (a 'group' is an analysis of identical markers).
- [x] All plots are contour plots with outliers or pseudocolor plots.
- [x] A numerical value for number of cells or percentage (with statistics) is provided.

Methodology

Sample preparation	The control and KO cells lines were labeled with the cell proliferation tracer carboxyfluoroscein succinimidyl ester (CFSE) (Biolegend). 125 000 cells were resuspended in 1ml PBS and 1ml of PBS containing 10M of CFSE was added. The cells were incubated at 37°C for 20 minutes and the incorporation was stopped by adding 10ml of complete DMEM as described above. Following centrifugation, the supernatant was removed and the cells were resuspended in complete DMEM, incubated for 10 minutes at room temperature and the cells were seeded in 60 mm dishes. At each time point (24, 48, 72 and 96 hours), the cells were harvested using 500ul of trypsin followed by two washes with ice-cold PBS. The cells were fixed in 100% ethanol and conserved at -20°C.
Instrument	The quantification of CFSE was measured by flow cytometry (BD Fortessa cytometer, Becton Dickinson)
Software	The analysis was achieved with the FlowJo software (LLC).
Cell population abundance	Not for CFSE cell count, as this is not a cell population distribution.
Gating strategy	Figure 4d includes an example of the gating strategy and the boundaries are described in the figure legend.

- [x] Tick this box to confirm that a figure exemplifying the gating strategy is provided in the Supplementary Information.
